# Effects of dietary salt on gene and protein expression in brain tissue of a model of sporadic small vessel disease

**DOI:** 10.1042/CS20171572

**Published:** 2018-06-26

**Authors:** Emma L. Bailey, Martin W. McBride, John D. McClure, Wendy Beattie, Delyth Graham, Anna F. Dominiczak, Colin Smith, Joanna M. Wardlaw

**Affiliations:** 1 School of Life Sciences, Thomson Building, University of Glasgow, University Avenue, Glasgow G12 8QQ, U.K.; 2 Institute of Cardiovascular and Medical Sciences, University of Glasgow, University Avenue, Glasgow G12 8QQ, U.K.; 3 Academic Department of Neuropathology, Centre for Clinical Brain Sciences, Little France, Edinburgh EH16 4SB, U.K.; 4 Centre for Clinical Brain Sciences, and UK Dementia Research Institute at the University of Edinburgh, Chancellors Building, 49 Little France Crescent, Edinburgh EH16 4SB, U.K.

**Keywords:** Salt, Small Vessel, stroke, SHRSP, White Matter

## Abstract

Background: The effect of salt on cerebral small vessel disease (SVD) is poorly understood. We assessed the effect of dietary salt on cerebral tissue of the stroke-prone spontaneously hypertensive rat (SHRSP) – a relevant model of sporadic SVD – at both the gene and protein level. Methods**:** Brains from 21-week-old SHRSP and Wistar-Kyoto rats, half additionally salt-loaded (via a 3-week regime of 1% NaCl in drinking water), were split into two hemispheres and sectioned coronally – one hemisphere for mRNA microarray and qRT-PCR, the other for immunohistochemistry using a panel of antibodies targeting components of the neurovascular unit. Results**:** We observed differences in gene and protein expression affecting the acute phase pathway and oxidative stress (*ALB, AMBP, APOH, AHSG* and *LOC100129193*, up-regulated in salt-loaded WKY versus WKY, >2-fold), active microglia (increased Iba-1 protein expression in salt-loaded SHRSP versus salt-loaded WKY, p<0.05), vascular structure (*ACTB* and *CTNNB*, up-regulated in salt-loaded SHRSP versus SHRSP, >3-fold; *CLDN-11, VEGF* and *VGF* down-regulated >2-fold in salt-loaded SHRSP versus SHRSP) and myelin integrity (*MBP* down-regulated in salt loaded WKY rats versus WKY, >2.5-fold). Changes of salt-loading were more pronounced in SHRSP and occurred without an increase in blood pressure in WKY rats. Conclusion: Salt exposure induced changes in gene and protein expression in an experimental model of SVD and its parent rat strain in multiple pathways involving components of the glio-vascular unit. Further studies in pertinent experimental models at different ages would help clarify the short- and long-term effect of dietary salt in SVD.

## Introduction

The association between high dietary salt intake and stroke incidence and mortality is well known [1,2]. However, the true interaction between salt intake, blood pressure and cerebrovascular disease (CVD) remains incompletely understood. Literature has begun to highlight a possible mechanism of salt independent of, or only partially mediated by, hypertension [1]. For example, epidemiology evidence suggests salt intake is associated with increased stroke risk and CVD independent of blood pressure (BP) [3]; however the present study, like many others, was conducted in a general (heterogeneous) stroke population and the effect of salt may differ across stroke subtypes. Recently, a study of minor ischaemic patients found an association between increased dietary salt intake and greater volume of white matter hyperintensities (WMH), the most frequent feature of small vessel disease (SVD), independent of BP or history of hypertension [4]. Long-term high dietary salt intake has also been positively associated with worse SVD features on neuroimaging, including lacunes, microbleeds, severe WMH’s and worse total SVD scores in patients with lacunar versus cortical stroke [5].

A major drawback of clinical studies is the inability to assess accurately patients’ salt intake. Experimental models provide an opportunity to do this in a controlled environment. Indeed a recent study has demonstrated that excess dietary salt suppresses resting cerebral blood flow and endothelial function, leading to cognitive impairment in mice – mechanisms and end stage outcomes relevant to SVD [6].

The spontaneously hypertensive stroke-prone rat (SHRSP) is considered to be a relevant model of sporadic SVD [7,8], but most studies investigating salt use in this strain have focused on kidney disorders or malignant hypertension with sparse investigation of cerebral tissue, particularly in relation to SVD.

We aimed to characterize genetic and protein changes in cerebral tissue of SHRSP rats compared with the parent WKY rats, with and without added dietary salt, to determine the impact of salt on the glio-vascular unit changes that are characteristic of SVD.

## Methods

For full methods see Supplementary Information.

### Animals

All animals were kept and experiments conducted according to U.K. regulations for live animal research in licenced laboratories (licence No. 60/3618) and the ARRIVE (Animal Research: Reporting In Vivo Experiments) guidelines (http://www.nc3rs.org/ARRIVE). All animals were obtained from the Glasgow colony and kept in identical conditions [9].

Study animals consisted of male rats aged 21 weeks reared on either a normal diet (*n* = 5 per strain) or a normal diet until age 18 weeks plus ‘salt-loading’ via 1% NaCl added to drinking water from 18 to 21 weeks (*n* = 5 per strain).

Tail cuff plethysmography was used to take weekly measurements of systolic BP. Animals were killed by overdose of isofluorane plus exsanguination. Brains were extracted and divided into left and right hemispheres – one fixed in formalin for immunohistochemistry, one snap frozen in liquid nitrogen for RNA extraction.

### Microarray and qRT-PCR

For mRNA analysis, one hemisphere was snap frozen and 2- mm coronal slices from a frontal and a mid-coronal region were cut using a Zivic® rat slicer matrix – these areas are typically the most damaged areas in SHRSP [9]. RNA was extracted using a Qiagen RNAeasy lipid tissue minikit (Qiagen Ltd., Manchester, U.K.) and transcribed to cRNA using an Ambion® Illumina® Total Prep RNA amplification kit (Applied Biosystems, Foster City, CA, U.S.A.). The resulting cRNA was loaded onto a RatRef12 microarray chip (Illumina, San Diego, CA, U.S.A.). Chips were scanned on an Illumina® Bead Reader (Illumina, San Diego, CA, U.S.A.) for fluorescence intensity. Samples were randomized throughout and were hybridized to chips and scanned at the same time. The same DNase-treated RNA was used as a template for synthesis of cDNA, qRT-PCR reactions using Applied Biosystems Taqman® Gene Expression Assay (Applied Biosystems, Foster City, CA, U.S.A.). The reaction mix included Taqman® universal master mix (Applied Biosystems, Foster City, CA, U.S.A.) plus GAPDH (VIC® labelled) and Taqman® probes corresponding to genes of interest (FAM® labelled). Results are reported according to the Minimum Information About a Microarray Experiment (MIAME) 2.0 criteria (http://www.mged.org/Workgroups/MIAME/miame_2.0.html).

### Immunohistochemistry

Formalin-fixed paraffin-embedded 3 mm frontal and mid coronal sections were cut into 7-µm sections. Antibodies assessed various components of the neurovascular unit (for full details of antibodies used see [9]), claudin-5, collagen IV, smooth muscle actin (SMA), collagen I, glial fibrillary acidic protein (GFAP), matrix metalloproteinase 9 (MMP9), ionized calcium-binding adaptor molecule 1 (Iba-1) and myelin basic protein (MBP). Antigen heat retrieval was performed before slides were blocked in hydrogen peroxide followed by either rabbit or swine serum. 3,3′-diaminobenzidine tetrahydrochloride with a haematoxylin counterstain revealed immunoreactivity. Tris-buffered saline replaced the primary antibody in negative controls.

### Data analysis

Microarray data were analysed using Rank Products (RP) analysis complete with Benjamini–Hochberg false discovery rate (FDR) adjustment. FDR  <0.05 was considered significant. A minimum individual fold change for significance was not set due to an interest in pathway interactions. Ingenuity Pathway Analysis® (IPA) (Ingenuity Systems, http://www.ingenuity.com) analysed data using both a pre-specified candidate gene approach and a genome-wide approach Significance of pathways was assessed using one-sided Fisher’s exact tests.

qRT-PCR data (cycle threshold (*C*_T_) values) were analysed in Microsoft® Excel by comparing mean delta cycle threshold (d*C*_T_) values versus the housekeeper gene using a Student’s *t*-test.

Percentage staining of immunohistochemistry was measured using ImagePro™ software (version 6.2; Media Cybernetics, Bethesda, MD, U.S.A.), blinded to species and age, in defined areas of cortical, deep grey and white matter. Statistical analysis was performed in Minitab using a general linear model (two-way ANOVA) followed by Tukey’s test for pairwise comparisons. *P* values of <0.05 were considered statistically significant. All data are shown as mean ± SEM.

## Results

Weekly systolic BP readings from 16 to 21 weeks were significantly higher in salt-loaded than in non-salt loaded SHRSP (average 237 ± 4 mmHg versus 200 ± 7 mmHg, *P*≤0.05). No difference in systolic BP was found between age-matched salt-loaded WKY versus WKY even at 21 weeks (154 ± 3 mmHg versus 152 ± 4 mmHg).

### Genetic data

#### Salt-loaded versus age-matched non-salt loaded animals – genome wide approach

##### Both strains

**Figure 1:**
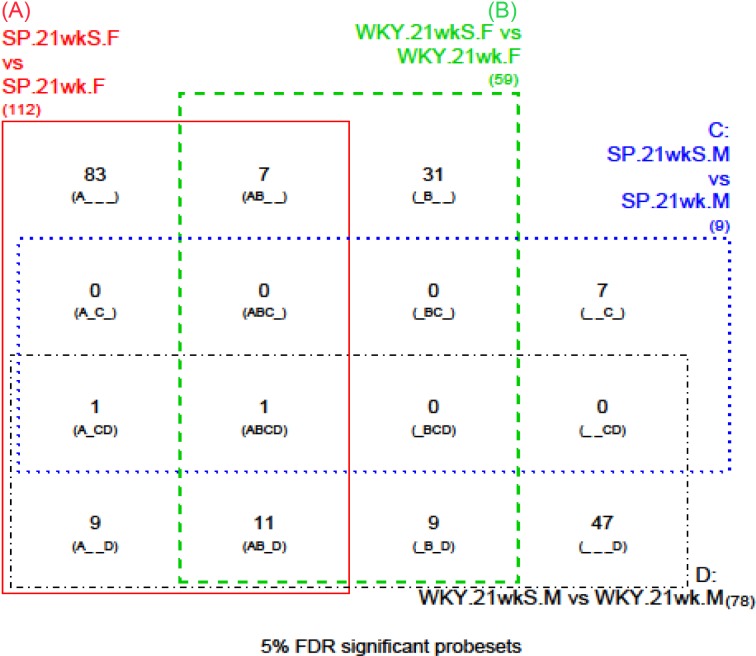
Four way Venn diagram from rank products analysis representing genes that were significantly differentially expressed in comparisons of salt (S) versus no salt animals, within (A) SHRSP (SP) frontal (F) brain sections; (B) WKY frontal brain sections; (C) SHRSP mid-coronal (M) brain sections; (D) WKY mid-coronal brain sections. The total number of significantly differentially expressed genes is given in brackets outside the relevant Venn rectangle (112 for A, 59 for B, 9 for C and 78 for D). Within the rectangles every possible combination of these sets of significant genes is given. For example, (A___) shows there were 83 genes that were only significant for the A comparison; (AB__) shows there were seven genes that were significant for both the A and B comparisons but not for C and D; (ABC_) shows there were 0 genes that were significant for the A, B and C comparisons but not for D; (ABCD) shows there was one gene significant for all four of the comparisons.

**Figure 2:**
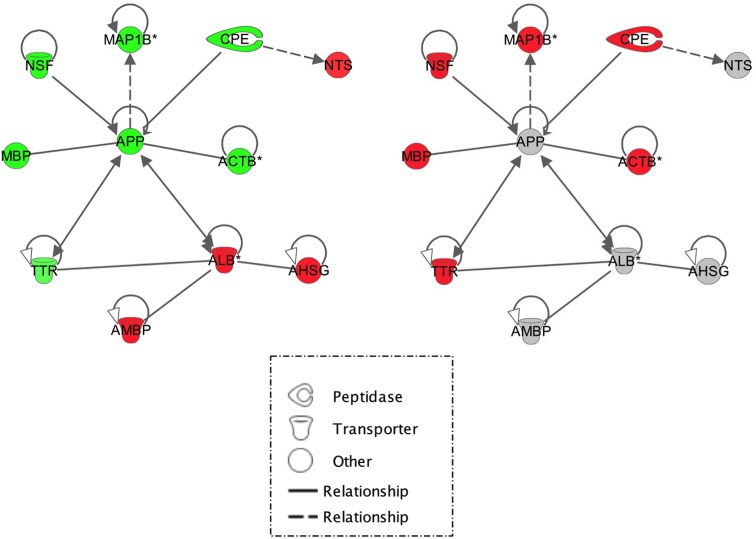
An IPA software network representing interactions between differentially expressed genes within frontal brain sections. Green, down-regulated genes and red, up-regulated genes. Statistics quoted are, from top to bottom – p value, fold change and signal intensity. Solid lines indicate direct interactions. Dotted lines indicate indirect interactions. (left) Salt-loaded versus non-salt loaded WKY. (right) The same network overlaid with salt-loaded versus non-salt loaded SHRSP data therefore genes highlighted in red indicate genes up-regulated in salt-loaded SHRSP compared with SHRSP. For full numerical data corresponding to this figure, see Supplementary Information.

**Figure 3:**
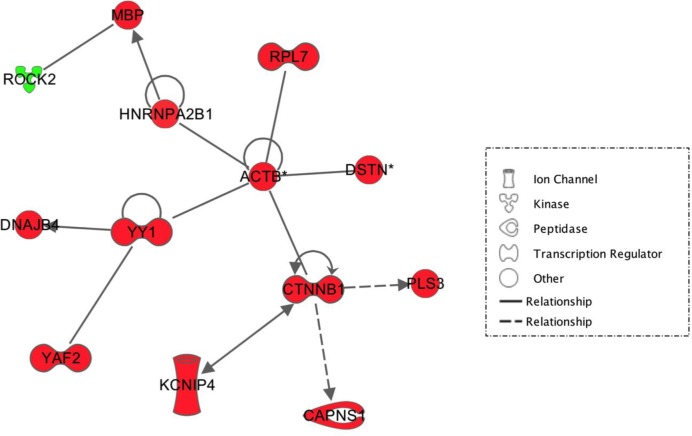
An IPA software network representing interactions between differentially expressed genes within the frontal sections of salt-loaded SHRSP versus non-salt loaded SHRSP. All genes highlighted in green are down-regulated in salt-loaded SHRSP. Genes highlighted in red are up-regulated in salt-loaded SHRSP. Statistics quoted are from top to bottom – p value, fold change and signal intensity. Solid lines indicate direct interactions. Dotted lines indicate indirect interactions. For full numerical data corresponding to this figure, see Supplementary Information.

In the frontal brain section, 59 genes were differentially expressed in salt-loaded WKY versus WKY (Figure 1). Within these, a small network of 11, centred on amyloid precursor protein (*APP*), showed down-regulation in salt-loaded WKY (Figure 2A). When salt-loaded SHRSP versus SHRSP data were overlaid, all of the genes surrounding *APP* (except albumin (*ALB*) and *APP* itself) were significantly up-regulated in the salt-loaded SHRSP suggesting a different response to salt-loading (Figure 2B). Within the frontal section, we also found a small network of genes centred round β-actin (*ACTB*) which were almost all up-regulated in salt-loaded SHRSP versus SHRSP (Figure 3), including some that are functionally related to maintaining the structural integrity of the vascular cytoskeleton (e.g. *ACTB*, Destrin (*DSTN*) and β-catenin (*CTNNB1*)).

#### The top ten up and down-regulated genes

##### Salt-loaded, WKY

The most up-regulated gene in both brain sections was *LOC100129193* (major urinary protein pseudogene), +16-fold in the frontal section and +4-fold in the mid-coronal section. The only gene to appear in the ten most highly up-regulated genes of both brain sections was *ALB* (+2-fold in both sections). In fact, a cluster of genes closely associated with *ALB* and the acute phase response pathway were up-regulated in the frontal section by at least 2-fold: α-1-microglobulin/bikunin precursor (*AMBP*), Apolipoprotein H (*APOH*) and α-2-HS-glycoprotein (*AHSG*). Transthyretin (*TTR*) – a transport protein implicated in amyloidosis – was also up-regulated in the mid-coronal section by 2-fold. The most down-regulated gene in salt-loaded versus non-salt loaded WKY was *MBP* in the frontal section (−2.6-fold). Only three other genes were highly down-regulated in both sections: *ACTB* down −2.3-fold and N-ethylmaleimide-sensitive factor (*NSF*) and M2-pyruvate kinase (*PKM2*) both down −2-fold Table 1.

**Table 1:** The top ten up- and down-regulated genes within and between strain and salt comparisons

	Frontal section				Mid-coronal section			
Salt	Up-regulated	Fold change	Down-regulated	Fold change	Up-regulated	Fold change	Down-regulated	Fold change
Salt-loaded WKY versus non-salt loaded WKY	LOC100129193 AMBP APOH ALDOB AHSG GC ALB GPM6A TCEB1 NTS	×16.3 ×2.6 ×2.2 ×2.2 ×2.2 ×2.0 ×2.0 ×1.9 ×1.9 ×1.8	MBP CPE ACTB TSPAN7 MAP1B PPP3CB TPI1 NSF PKM2 SETD3	×2.6 ×2.4 ×2.3 ×2.2 ×2.1 ×2.0 ×1.9 ×1.9 ×1.9 ×1.9	LOC100129193 TMEM27 SLCO1A5 GPM6A MCTP1 TAC1 GPR88 TTR LRRC7 ALB	×4.2 ×2.6 ×2.3 ×2.2 ×2.1 ×2.1 ×2.0 ×2.0 ×1.9 ×1.9	PRKCD ACTB WSF1 VWF NSF PKM2 SLC24A2 OXT VGF PRKCG	×2.5 ×2.3 ×2.3 ×2.3 ×2.2 ×2.1 ×2.1 ×2.1 ×1.9 ×1.9
Salt-loaded versus non-salt loaded SHRSP	TTR SOSTDC1 SLCO1A5 OTX2 CPE ACTB F5 GDI1 SNURF TSPAN7	×53.4 ×10 ×5.2 ×3.9 ×3.8 ×3.7 ×3.6 ×3.4 ×3.4 ×3.3	VOPP1 RPS13 TPI1 SEC61G ROCK2 CYR61 HBXIP MYEF2 GSK3B IL11	×2.7 ×2.1 ×2.1 ×1.9 ×1.9 ×1.9 ×1.8 ×1.8 ×1.8 ×1.7	LOC100129193 SLC17A6 SLC24A2 HBB OPCML SEPT7 AMBP CABP7 ALB ANXA1	×3.3 ×2.2 ×2.0 ×1.9 ×1.9 ×1.9 ×1.8 ×1.7 ×1.7 ×1.7	TTR SOSTDC1 ADORA2A AQP1 SCN4B SLC32A1 RASD2 DLX5 – –	×3.1 ×2.8 ×2.4 ×2.3 ×2.1 ×2.0 ×2.0 ×1.9 – –
Salt-loaded SHRSP versus salt-loaded WKY	RGD1564649 RSP9 GUCY1A3 TTR FAM151B RGD1311103 SOSTDC1 ZNF597 OXT RGD1566136	×46.1 ×19.5 ×15.3 ×7.2 ×5.8 ×5.6 ×5.0 ×4.4 ×3.2 ×3.0	MRPL18 HCG 2004593 LOC100125697 PXMP4 RGD1565336 GPR18 ALB C20ORF7 CSNK2A1 RGD1564078	×39.4 ×18.3 ×5.3 ×4.7 ×4.5 ×4.1 ×4.0 ×3.6 ×3.5 ×3.5	RGD1564649 RSP9 GUCY1A3 FAM151B RGD1311103 ZNF597 AVP PMCH SIPA1L2 RGD1566136	×43.9 ×16.8 ×14.5 ×8.0 ×6.1 ×4.7 ×3.5 ×3.3 ×2.9 ×2.8	MRPL18 HCG 2004593 RGD1565336 LOC100125697 GPR18 PXMP4 ALB HLA-C VPS13C CSNK2A1	×41.7 ×16.1 ×5.2 ×4.8 ×4.6 ×3.6 ×3.6 ×3.3 ×3.2 ×3.0

Results are for each brain section. *N* = 4 for all groups. All genes listed are significantly expressed when an FDR of *q*<0.05 is applied.

Abbreviations: ACTB, β-actin; ADORA2A, adenosine A2a receptor; AHSG, α-2-HS-glycoprotein; ALDOB, aldolase B, fructose-bisphosphate; AMBP, α-1-microglobulin/bikunin precursor; ANXA1, annexin A1; APOH, Apolipoprotein H; AQP1, aquaporin 1; SCN4B, sodium channel, voltage-gated, type IV, β; AVP, arginine vasopressin; CABP7, calcium binding protein 7; CPE, carboxypeptidase E; CSNK2A1, casein kinase 2, α 1 polypeptide; CYR61, cysteine-rich, angiogenic inducer, 61; DLX5, distal-less homeobox 5; F5, coagulation factor V; FAM151B, family with sequence similarity 151, member B; GC, group specific component; GDI1, GDP dissociation inhibitor 1; GPM6A, glycoprotein m6a; GPR18, G protein-coupled receptor 18; GPR88, G protein-coupled receptor 88; GSK3B, glycogen synthase kinase 3 β; GUCY1a3, guanylate cyclase α subunit 3; HBB, hemoglobin, β; HBXIP, hepatitis B virus x interacting protein; HLA-C, major histocompatibility complex, class C; IL11, interleukin 11; LOC100129193, major urinary protein pseudogene; LRRC7, leucine rich repeat containing 7; MAP1B, microtubule-associated protein 1B; MCTP1, multiple C2 domains, transmembrane 1; MRPL18, mitochondrial ribosomal protein L18; MYEF2, myelin expression factor 2; NSF, N-ethylmaleimide-sensitive factor; NTS, neurotensin; OPCML, opioid binding protein/cell adhesion molecule-like; OTX2, orthodenticle homeobox 2; OXT, oxytocin; PKM2, pyruvate kinase, muscle; PMCH, pro-melanin-concentrating hormone; PPP3CB, protein phosphatase 3, catalytic subunit, β isoform; PRKCG, protein kinase C, γ; PXPM4, peroxisomal membrane protein 4; RASD2, RASD family, member 2; RGD, Rat genome database; ROCK2, Rho-associated coiled-coil containing protein kinase 2; RSP9, ribosomal protein 9; SEC61G, SEC61, γ subunit; SEPT7, septin 7; SETD3, SET domain containing 3; SIPA1L2, signal-induced proliferation-associated 1 like 2; SLC17A6, solute carrier family 17 member 6; SLC24A2, solute carrier family 24 member 2; SLC32A1, solute carrier family 32 member 1; SLCO1A5, solute carrier organic anion transporter family, member 1a5; SNURF, SNRPN upstream reading frame; SOSTDC1, sclerostin domain containing 1; TAC1, tachykinin 1; TCEB1, transcription elongation factor B (SIII), polypeptide 1; TMEM27, transmembrane protein 27; TPI1, triosephosphate isomerase 1; TSPAN7, tetraspanin 7; TTR, transthyretin; VGF, vascular growth factor; VOPP1, vesicular, overexpressed in cancer, prosurvival protein 1; VPS13C, vacuolar protein sorting 13 -homologue; ZNF597, Zinc finger protein 597.

##### Salt-loaded, SHRSP

Here, there was much less consistency between brain sections. Like salt-loaded versus non-salt loaded WKY, *TTR* was up-regulated in the frontal section but this time by +53.4-fold. In contrast with the salt-loading WKY comparison above, *ACTB* was up-regulated in the frontal section (+3.7-fold). In the mid-coronal section *LOC100129193* was the most up-regulated (+3.3-fold) similar to salt-loaded versus non-salt loaded WKY. Here, there were also two solute carriers *SLC17a6* (+2.2-fold) and *SLC24a2* (+2.0-fold) as well as *ALB* (+1.7-fold) which were up-regulated. In the frontal section, no gene was substantially down-regulated, but several were down-regulated by approximately a −2-fold, topped by *VOPP1* (vesicular, overexpressed in cancer, pro-survival protein 1) with a fold change of −2.7. The remaining genes all had fold changes less than 2 and the only other gene of note was *CYR61*, an angiogenic inducer (down-regulated −1.9-fold). In the mid-coronal section, only eight genes were significantly down-regulated with salt-loading in the SHRSP, of which *TTR* came top with a fold change of −3.1, which directly contrasts the up-regulation seen in the frontal section of salt-loaded SHRSP versus SHRSP and the mid-coronal section of salt-loaded WKY versus WKY. This was followed by Von Willebrand factor (*VWF*; −2.3-fold) and vascular growth factor (*VGF*; −1.9-fold).

##### Salt-loaded SHRSP versus WKY

Between the two salt-loaded strains the most differentially expressed genes were representative of those previously found at ages 5 and 16 weeks in non-salt loaded WKY and SHRSP [10]. *GUCY1a3* (guanylate cyclase soluble subunit α-3), *RSP9* (repeat sequence probe 9) and *RGD1564649* (similar to 40S ribosomal protein S9) were up-regulated in SHRSP by at least +14-fold in both brain sections, whilst *MRPL18* (mitochondrial ribosomal protein L18), *HCG2004593* (ribosomal protein L17 pseudogene 39) and *LOC100125697* (low-molecular-weight glutenin storage protein) were down-regulated by a minimum of −5-fold in both brain sections. *ALB* was also down-regulated in both brain sections by at least −3.5-fold.

#### Analysis of over-represented biological pathways and genes within biological pathways of interest on ingenuity pathway analysis (Figure 4)

Tight junctions (blood–brain barrier) [9,11,12] and the acute phase response (a rapid inflammatory response that provides protection against microorganisms using non-specific defence mechanisms) [13] are considered to be affected in cerebral SVD, so we looked for genes of interest which were differentially expressed within these pathways.

**Figure 4:**
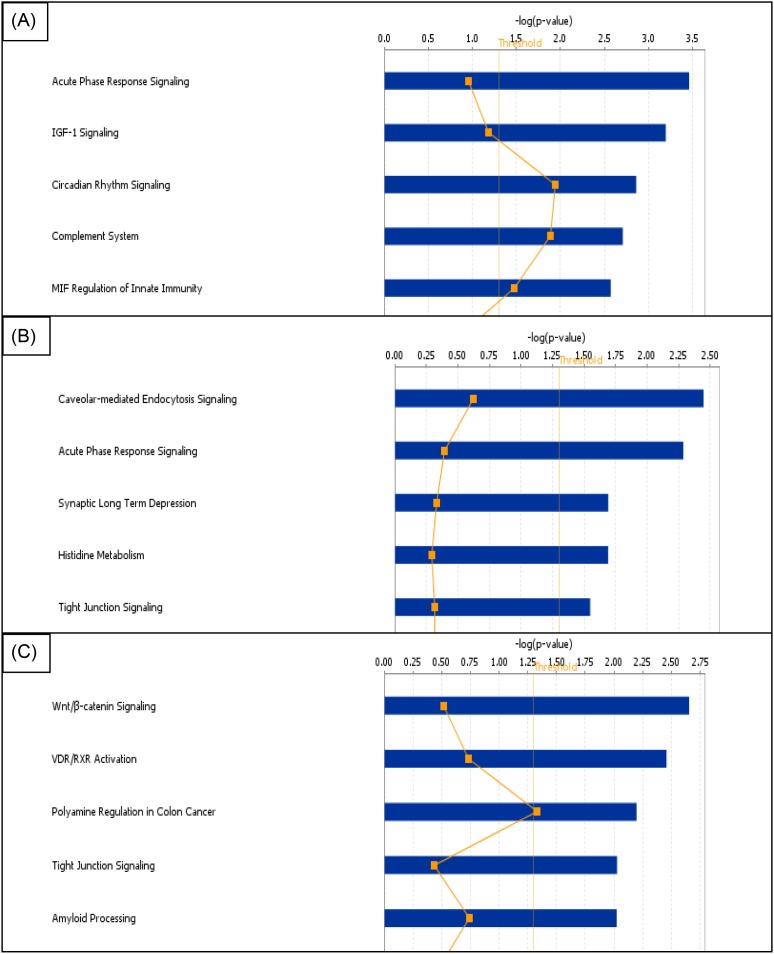
The top five biological pathways in IPA containing an over-representation of significantly differentially expressed genes in the frontal sections of (A) salt-loaded WKY versus non-salt loaded WKY, (B) salt-loaded SHRSP versus salt-loaded WKY and (C) salt-loaded SHRSP versus non-salt-loaded SHRSP. The blue bar represents how significant the uploaded data set is to the pathway (log *P* value) and the orange line represents the number of genes differentially expressed as a proportion of the total genes within that pathway.

The acute phase response signalling pathway contained the most differentially expressed genes in both rat strains for all salt versus no salt comparisons, consistently appearing within the top five affected pathways. Salt-loading in both rat strains was associated with changes within the tight junction signalling pathway of frontal brain sections. Pathways pertaining to oxidative stress and leucocyte extravasation also contained a high proportion of differentially expressed genes in both strains with salt-loading versus non-salt loading particularly in the frontal brain sections.

In the frontal section of salt-loaded versus not-salt loaded SHRSP, we found Claudin-11 (*CLDN11*) to be down-regulated −2.3-fold within the tight junction signalling pathway. However, we also found *CLDN11* to be up-regulated in salt-loaded SHRSP when compared with salt-loaded WKY (+1.8-fold change) suggesting a difference in response to salt between the strains.

In the acute phase response pathway, *ALB* was consistently up-regulated across all salt comparisons in both strains. Additionally, in WKY in the frontal section, a group of closely associated genes *AMBP, AHSG* and *APOH* were up-regulated by approximately 2-fold with salt-loading.

#### qRT-PCR of candidate genes

From the microarray results above, we chose the following genes for quantitative validation with qRT-PCR: MBP, ACTB and SLC24a2. MBP was chosen due to down-regulation (> −2-fold) in the frontal section of salt-loaded WKY versus non-salt loaded WKY and any subsequent effect on protein expression could be directly assessed via immunohistochemistry. ACTB was chosen due to significant down-regulation in salt-loaded WKY versus non-salt loaded WKY in both brain sections (> −2-fold) whilst also showing a significant up-regulation in the frontal section of salt-loaded SHRSP versus non-salt loadedSHRSP (+3.7-fold). SLC24a2 was chosen because, like ACTB, the microarray data showed two significant results. Firstly, SLC24a2 was significantly down-regulated in both sections of salt-loaded WKY versus non-salt loaded WKY (−2-fold). Secondly, we found SLC24a2 to be significantly up-regulated in the mid-coronal section of salt-loaded SHRSP (+2-fold) versus non-salt loaded SHRSP. Unfortunately, qRT-PCR did not replicate any of the significant differences in the three genes of interest.

#### Immunohistochemistry data

For full numerical immunohistochemical data see Supplementary Tables S1–S4.

##### Salt-loading, WKY

**Figure 5:**
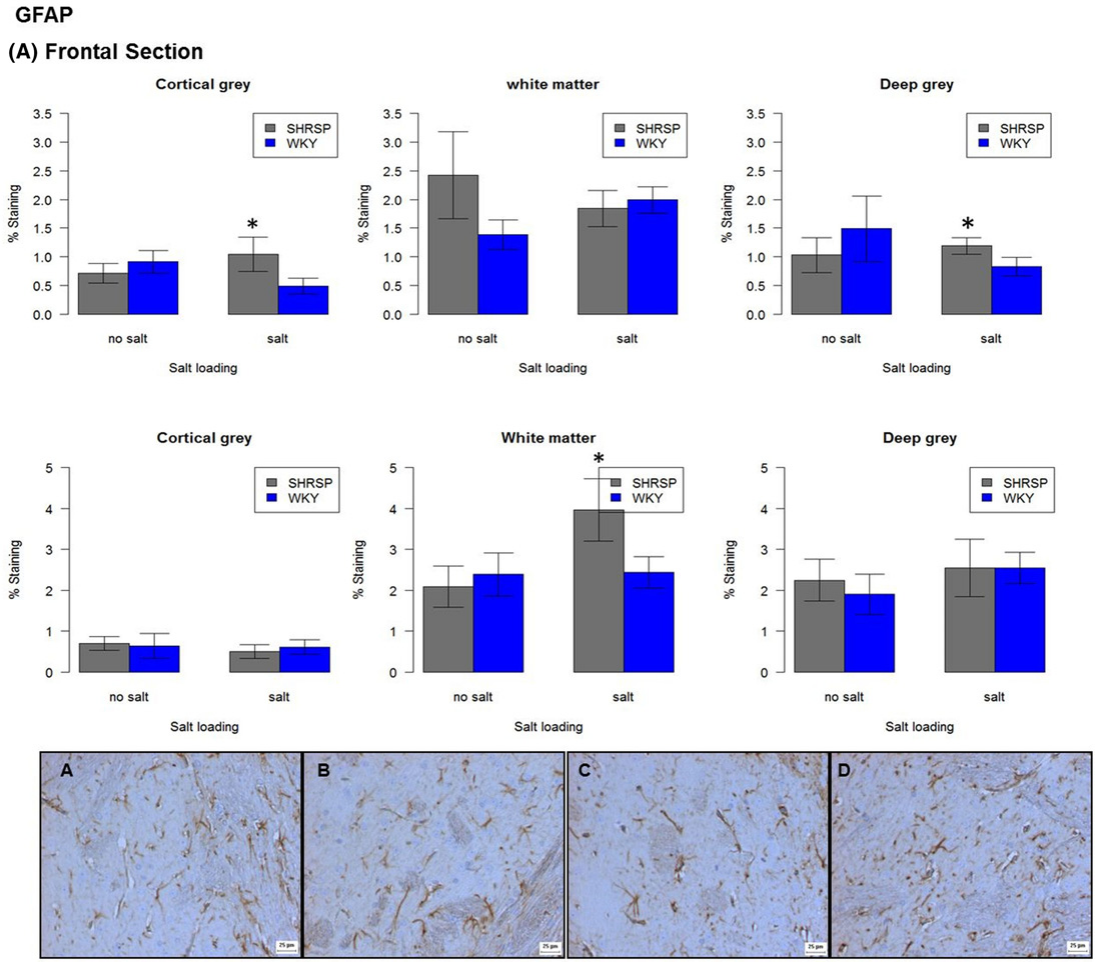
Immunoreactivity of GFAP in frontal and mid-coronal sections non-salt loaded versus salt-loaded SHRSP and WKY. Each bar represents *N*=5. Error bars represent standard error of the mean. Staining panel (**A**) non-salt loaded WKY, (**B**) salt-loaded WKY, (**C**) non-salt loaded SHRSP and (**D**) salt-loaded SHRSP. All images taken at ×10 objective in the deep grey matter of a frontal section. *P<0.05

**Figure 6:**
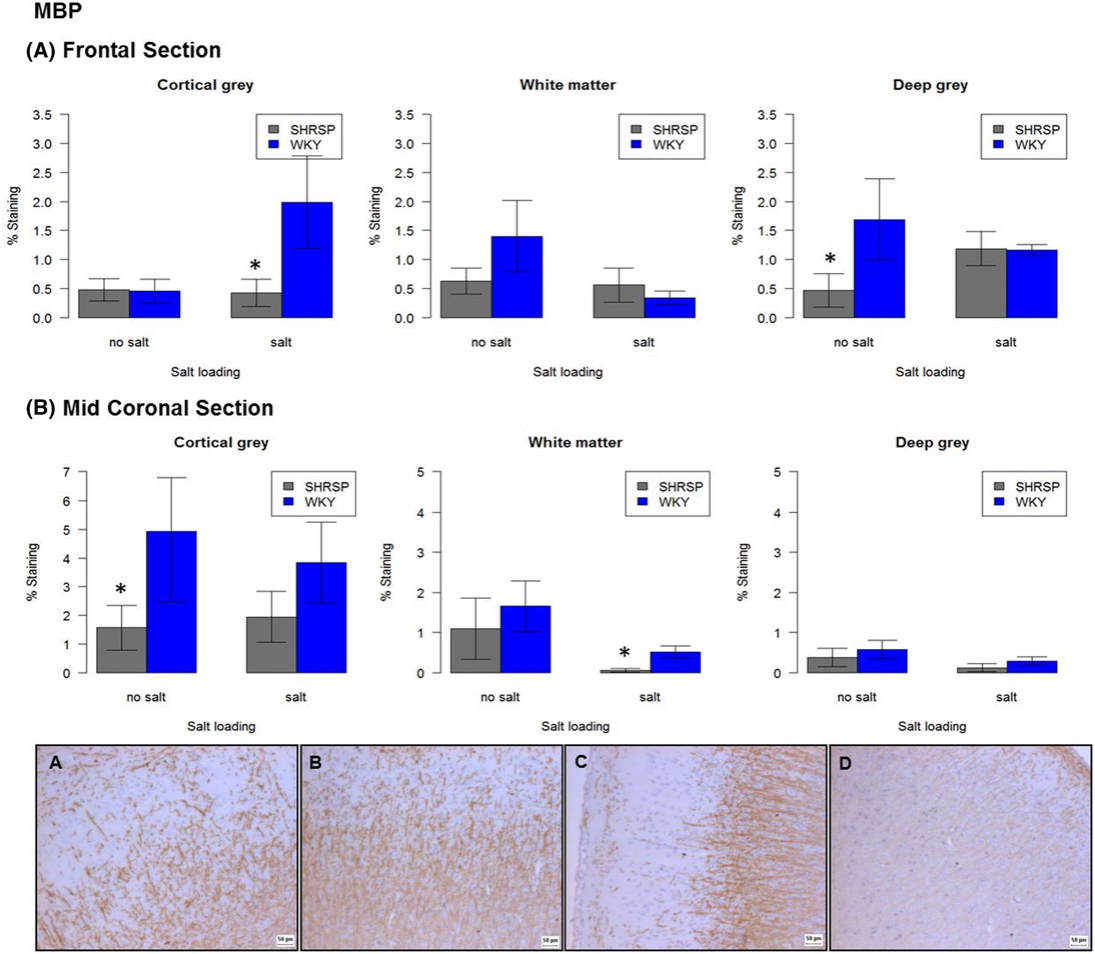
Immunoreactivity of MBP in frontal and mid-coronal sections of non-salt loaded versus salt-loaded SHRSP and WKY. Each bar represents *N*=5. Error bars represent standard error of the mean. Staining panel (**A**) non-salt loaded WKY, (**B**) salt-loaded WKY, (**C**) non-salt loaded SHRSP and (**D**) salt-loaded SHRSP. All images taken at ×4 objective in the cortical grey matter of a frontal section. *P<0.05.

In WKY, salt-loading was associated with reduced GFAP immunoreactivity in the frontal section of cortical grey matter (*P*<0.05) (Figure 5). Claudin-5 immunoreactivity was reduced in all areas particularly in the frontal cortex, although this did not reach statistical significance. MBP immunoreactivity (Figure 6) was significantly increased in the cortical grey matter of the frontal section (*P*=0.01), whilst significantly decreased in the white matter of the same section (*P*<0.05). MBP in the white matter of the mid-coronal section was significantly increased (*P*<0.05).

##### Salt-loading, SHRSP

In SHRSP, salt-loading was associated with decreased Collagen IV in the cortical grey matter of the frontal section (*P*<0.05). Iba-1 expression varied greatly within the cortical grey matter of the two brain sections – it was significantly lower in the cortical grey matter of the frontal section (*P*<0.01), but significantly higher in cortical grey matter of the mid-coronal section (p = 0.01). GFAP immunoreactivity was significantly increased in the white matter of the mid-coronal section (p<0.01) in salt-loaded SHRSP versus non-salt loaded SHRSP (Figure 5). Salt-loaded SHRSP tended to have less SMA immunoreactivity across all regions in both frontal and mid-coronal sections although this did not reach statistical significance. MBP immunoreactivity was significantly decreased in salt-loaded SHRSP in the white matter of the mid-coronal section (p<0.01) (Figure 6).

##### Salt-loading, WKY versus SHRSP

Salt-loading obscured some between-strain differences seen in rats fed normal chow [9], and exaggerated others. For example, we did not see the difference in Claudin-5 immunoreactivity previously found between WKY and SHRSP rats fed a normal diet repeated in the salt-loaded animals, as the amount of Claudin-5 immunoreactivity in salt-loaded WKY rats had also decreased. GFAP immunoreactivity was significantly increased in the cortical and deep grey matter of the frontal section (both *P*<0.05) and the white matter of the mid-coronal section (*P*<0.01) of salt-loaded SHRSP versus salt-loaded WKY (Figure 5). Iba-1 immunoreactivity was no different between salt-loaded strains in the frontal brain section; however, in the mid-coronal section salt-loaded SHRSP had significantly increased Iba-1 immunoreactivity in the cortical grey and deep matter compared with salt-loaded WKY (*P*<0.05 and *P*=0.01 respectively).

#### Assessment of gene expression corresponding to proteins assessed using immunohistochemistry

Of the proteins assessed using immunohistochemistry, only the gene for *MBP* was differentially expressed. In salt-loaded WKY versus non-salt loaded WKY, *MBP* was down-regulated in the frontal section (−2.6-fold), immunohistochemistry showed protein levels of MBP to be significantly more in the cortical grey matter of the frontal section (1.99 versus 0.47%, *P*<0.01) whilst being significantly decreased in the white matter of both frontal (0.35 versus 1.4%, *P*<0.05) and mid-coronal sections (0.521 versus 1.66%, *P*<0.01) of salt-loaded WKY versus non-salt loaded WKY (Figure 6).

In salt-loaded versus non-salt loaded SHRSP, *MBP* was up-regulated by +2.2-fold in the frontal section. Immunohistochemistry showed protein levels of MBP to be significantly increased in the frontal deep grey matter (1.19 versus 0.47%, *P*<0.01), whilst being significantly decreased in the mid-coronal white matter (0.06 versus 1.1%, *P*<0.05) thereby agreeing with salt-loaded WKY versus non-salt loaded WKY. There was no difference in *MBP* gene expression between salt-loaded WKY and salt-loaded SHRSP; however, immunohistochemistry showed a significant decrease in MBP protein levels in the cortical grey matter of the frontal section (0.43 versus 1.99%, *P*<0.01) and the white matter of the mid-coronal section (0.06 versus 0.52%, *P*<0.05) (Figure 6).

## Discussion

**Figure 7:**
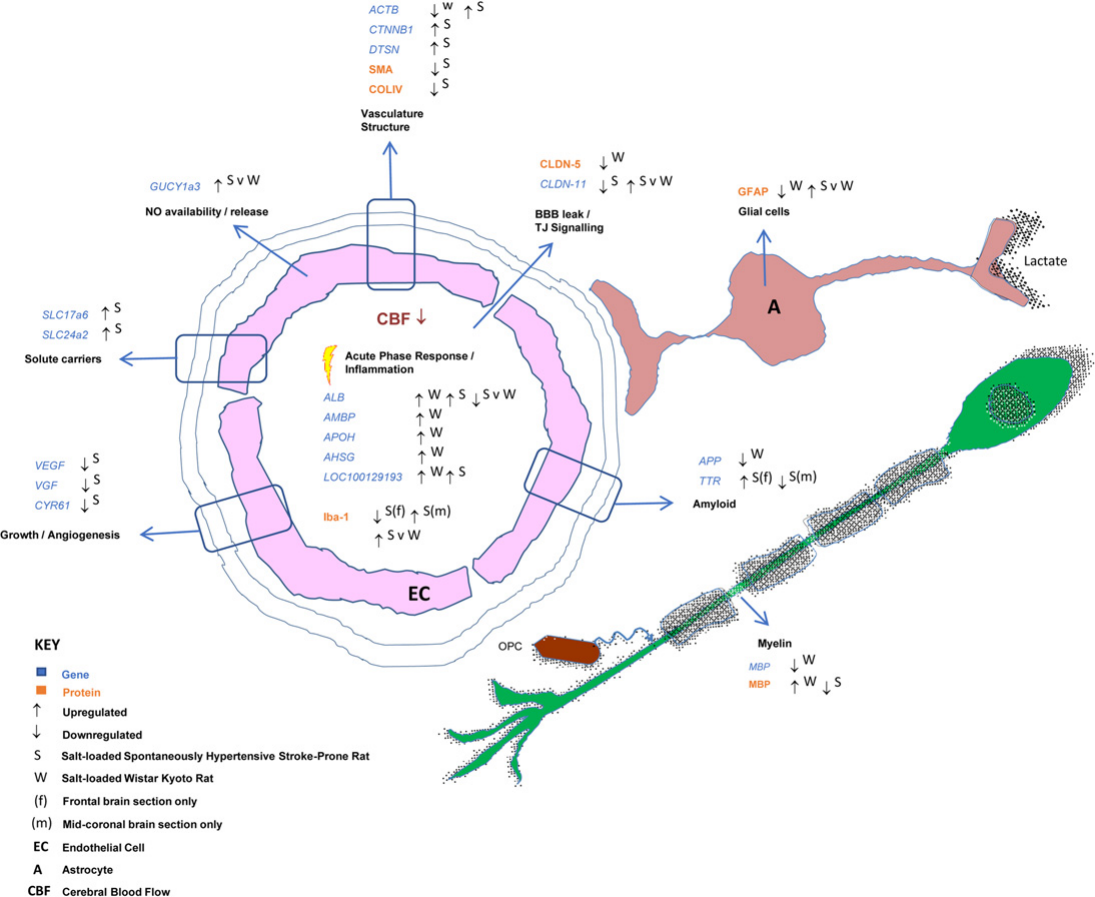
A diagrammatical summary of both genetic and protein changes affecting the neurovascular unit observed in the cerebral tissue of salt-loaded WKY and SHRSP rats.

The present study has highlighted changes with dietary salt-loading in gene and protein expression in networks affecting inflammatory pathways, vascular structure and myelin integrity in the brains of SHRSP, a sporadic model of SVD, and in its parent strain, the WKY rat (for a diagrammatic summary see Figure 7). In salt-loaded WKY rats, these changes occurred independently of an increase in blood pressure. Taken together, these results suggest that additional dietary salt may produce tissue changes indicative of SVD-related damage that are at least in part independent of the level of blood pressure.

Whilst the present study consisted of a relatively small sample size and a short duration of salt-loading (3 weeks), rats were randomly selected from the Glasgow colony for both the control and salt-loaded groups, careful blinding was undertaken and only established validated techniques were used. Both protein and gene expression changes were assessed in the same animals, reducing between-animal noise and confirming previously reported genetic strain differences between WKY and SHRSP [10] including the up-regulation of *GUCY1a3* and down-regulation of *MRPL18* and *ALB*. These between-strain differences – which represent changes associated with the nitric oxide, inflammatory and connective tissue pathways – were larger in magnitude than the effects of salt-loading indicating the effect of rat strain is dominant. However, the fact that differences were found with a short period of salt-loading in both strains cannot be ignored and whilst the majority of changes in gene expression were small in terms of fold change (meaning confirmation with PCR was difficult), in combination and in the long term they could be extremely detrimental. Indeed a recent study in mice has implicated excess salt in endothelial dysfunction caused via inhibition of eNOS phosphorylation and reduced production of NO [6]. In turn this resulted in cognitive deficits in the salt-fed mice when tested for spatial memory, novel object recognition and nesting behaviour.

Pathways pertaining to oxidative stress and inflammation showed changes in gene expression due to salt-loading in both strains. Acute phase response signalling contained the most differentially expressed genes in all salt versus no salt comparisons, whilst pathways pertaining to oxidative stress and leucocyte extravasation were also significantly affected. Increased expression of *ALB* and decreased expression of *LOC100129193* have previously been implicated in hyperhomocysteinemia [14]; however, in both salt-loaded WKY and SHRSP this study saw an increase in *LOC100129193* and a decrease in *ALB*. This disagreement suggests salt-loading may influence inflammatory pathways over different time periods or durations of exposure or that they may not act via homocysteine.

*TTR* expression was highly influenced by dietary salt in SHRSP being up-regulated by over +50-fold in frontal brain sections (though down-regulated in mid-coronal sections), and being amongst the ten most up-regulated genes in the salt-loaded WKY versus non-salt loaded WKY. *TTR* was also 7-fold more up-regulated in the salt-loaded SHRSP versus salt-loaded WKY whereas it was only up-regulated by 2-fold in the mid-coronal section of salt-loaded WKY versus non-salt loaded WKY and therefore did not make the top 10. *TTR* has been previously shown to have associations with senile systemic amyloidosis and plasma cell dyscrasias providing some evidence of a direct linkage to the immune response and potentially to the dementia-related effects of SVDs [15].

In salt-loaded SHRSP at the protein level, we found increased reactive gliosis and activation of microglial cells compared with salt-loaded WKY. These results agree with previous data showing increased levels of oxidative stress and inflammation to be established responses to excessive salt intake in SHRSP [16,17]. Salt-loading also resulted in down-regulation of a gene network involved in maintaining vascular structure and tight junction signalling featured in the top 5 pathways affected in both salt-loaded WKY and SHRSP. Within the tight junction signalling pathway, we found *CLDN11* gene expression was significantly down-regulated in salt-loaded versus non-salt-loaded SHRSP and conversely up-regulated in salt-loaded SHRSP compared with salt-loaded WKY. As the SHRSP already has reduced Claudin-5 protein expression independent of salt-loading [9], the difference in Claudin-11 could indicate increased leakiness of blood vessels particularly when coupled with changes in gene expression of albumin. The difference in immunoreactivity of Claudin-5 between the strains at 21 weeks of age was absent in salt-loaded animals because the salt-loaded WKY rats also had less Claudin-5. These results suggest that WKY also develop vascular damage in the presence of salt-loading. The changes in *APP* and *MBP* as well as *VWF* and *VGF* in salt-loaded WKY versus WKY could indicate further impairments to vascular structure, and underline the importance of avoiding salt-loading in studies of blood–brain barrier integrity.

Furthermore, salt-loading caused differential expression of genes associated with myelin integrity in both strains. In WKY rats, several genes in a network centred round *APP* and *MBP* were up-regulated suggesting that expression of myelin-associated proteins would be increased. MBP immunoreactivity was also affected by salt-loading in WKY rats, although the changes were somewhat erratic with expression both increasing and decreasing. As well as involvement in myelination, MBP is directly involved in T-lymphocyte mediated inflammation which can also lead to increased permeability of the blood-brain barrier (BBB) [13]. Indeed many studies have suggested an inflammatory mechanism in salt-loaded rats which in turn compromises the blood–brain barrier [11,12]. Genes centred round *APP* and *MBP* were down-regulated in salt-loaded versus non-salt loaded SHRSP and we also saw decreased MBP immunoreactivity with salt-loading in SHRSP. This suggests a different response to salt in WKY versus SHRSP or could represent a different stage in a disease process and requires further investigation.

The SHRSP is considered a relevant model of sporadic SVD in its naïve state [7]. SHRSP develop high levels of hypertension with systolic pressures of over 170 mmHg in contrast with normal levels of systolic pressure in WKY of 130–140 mmHg [18]. Salt-loaded SHRSP regularly display systolic blood pressures of over 200 mmHg and accelerated end stage pathology [19] meaning they rarely live beyond 28 weeks of age. In the SHRSP, solute carriers such as *SLC24a2* (a sodium/potassium/calcium exchanger) were frequently within the top 10 up- or down-regulated genes. The fact that these genes were affected in SHRSP and not WKY provide further evidence of the salt sensitivity of the SHRSP [20].

A recent translational study tested associations between genes differentially expressed in the brains of young SHRSP and human WMH [21] and, despite these genes having small fold changes individually, collectively they showed positive associations with human WMH, consistent with the hypothesis that WMH are multi-factorial in nature and have a vascular component. The genes identified in the present study should therefore also be tested in clinical cohorts of small vessel disease patients to see if the effect of dietary salt translates.

## Conclusion

The present study showed changes in both gene and protein expression in the cerebral tissue of salt-loaded WKY independent of changes in BP. In both strains, the cerebral changes are exacerbated by salt-loading, although the effect is more pronounced in the SHRSP. Studies to assess different durations of salt-loading in younger non-hypertensive animals are required and salt intake should be further assessed in clinical studies of SVD. Studies using the SHRSP as a model of cerebral SVD should refrain from salt-loading their animals to prevent complicating an already intricate set of pathological changes.

## Clinical perspectives

- The effect of salt on cerebral small vessel disease (SVD) is poorly understood and dietary salt is difficult to measure and control in the clinic. Therefore, the spontaneously hypertensive stroke-prone rat – a relevant experimental model was used to assess the effects of dietary salt on cerebral gene and protein expression.
- Dietary salt-loading caused changes in gene and protein expression in networks affecting inflammatory pathways, vascular structure and myelin integrity in the brains of SHRSP, and in its parent strain, the WKY rat. In the latter changes occurred independently of blood pressure elevation.
- The genes identified in the present study should be tested in clinical cohorts of small vessel disease patients to see if the effect of dietary salt translates from bench to bedside.

## Supporting information

**Table S1.** Results of ANOVA analysis on the effect of salt on the percentage staining of 4 antibodies assessing vascular structure

**Table S2.** Results of ANOVA analysis on the effect of salt on the percentage staining of 4 antibodies assessing vascular structure

**Table S3.** Results of ANOVA analysis on the effect of salt on the percentage staining of 4 antibodies assessing the presence / absence of vascular disease

**Table S4.** Results of ANOVA analysis on the effect of salt on the percentage staining of 4 antibodies assessing the presence / absence of vascular disease

## Abbreviations

BP: blood pressure
*C*_T_: cycle threshold
CVD: cerebrovascular disease
FDR: false discovery rate
qRT-PCR: quantitative real-time polymerase chain reaction
RP: rank products
SHRSP: spontaneously hypertensive stroke-prone rat
SVD: small vessel disease
WKY: Wistar-Kyoto rat
WMH: white matter hyperintensities
