# Supplementary material for: Effects of dietary salt on gene and protein expression in brain tissue of a model of sporadic small vessel disease
Source: Clin Sci (Lond). 2018 Jun 26;132(12):1315–28. doi: 10.1042/CS20171572 (PMC6365623; doi:10.1042/CS20171572)
Supplement: Supplementary file 1 [file cs-132-cs20171572_supp1.pdf]

METHODS

Animals

All animals were kept and experiments conducted according to UK regulations for live animal research in licensed laboratories (licence No. 60/3618) and conducted according to the ARRIVE (Animal Research: Reporting In Vivo Experiments) guidelines (http://www.nc3rs.org/ARRIVE). Results are reported according to the Minimum Information About a Microarray Experiment (MIAME) 2.0 criteria (http://www.mged.org/Workgroups/MIAME/miame_2.0.html). All animals were obtained from the Glasgow colony and kept in identical conditions [8].

Study animals consisted of male rats aged 21 weeks reared on either a normal diet (*n* = 5 per strain) or a normal diet until age 18 weeks and then a 'salt-loaded' diet consisting of 1% NaCl added to drinking water from 18 to 21 weeks (*n* = 5 per strain).

Tail cuff plethysmography was used to take weekly measurements of systolic blood pressure. Animals were sacrificed by overdose of isofluorane plus exsanguination. Brains were extracted and divided into left and right hemispheres – one fixed in formalin for immunohistochemistry, one snap frozen in liquid nitrogen for RNA extraction.

Microarray

For full methods see [9].For mRNA analysis, one hemisphere was snap frozen in RNA-later ice solution and 2 mm coronal slices from a frontal and a mid-coronal region were cut using a Zivic® rat slicer matrix. One region was selected at +1.8-mm bregma to capture a representative frontal section and a second region at −1.72-mm bregma to capture representative areas of the basal ganglia and internal capsule (mid-coronal section). These

areas are typically affected in patients, and, from the published literature, typically are the most damaged areas in SHRSP [8].

RNA was extracted from homogenized coronal slices using a Qiagen RNAeasy lipid tissue minikit (Qiagen Ltd., Manchester, UK). Turbo DNase removed any remaining genomic DNA and RNA quality was assessed on a Nanodrop 1000 and Agilent® bioanalyser 2100 (Agilent, Santa Clara, CA, USA). RNA was transcribed to cRNA using an Ambion® Illumina® Total Prep RNA amplification kit (Applied Biosystems, Foster City, CA, USA). We obtained a final cRNA elute of ~200µl and checked cRNA quality on an Agilent® bioanalyser. The resulting cRNA was loaded onto a RatRef12 microarray chip (Illumina, San Diego, CA, USA), containing 22 519 gene and probe sets and stained in a solution of E1 buffer plus 1:1000 dilution of streptavidin-Cy3. Chips were scanned on an Illumina® Bead Reader (Illumina, San Diego, CA, USA) for fluorescence intensity. Samples with a signal intensity of >600 passed the bead array reader's quality control. We randomized samples throughout the entire microarray protocol and all samples were hybridized to the chips and scanned at the same time.

qRT-PCR: To quantitatively confirm differential expression from the microarray the same DNase-treated RNA was used as a template for synthesis of cDNA, qRT-PCR reactions using Applied Biosystems Taqman® Gene Expression Assay (Applied Biosystems, Foster City, CA, USA). The reaction mix included Taqman® universal master mix (Applied Biosystems, Foster City, CA, USA) plus GAPDH (VIC® labelled)] and Taqman® probes corresponding to our gene of interest (FAM® labelled).

Data Analysis: Microarray data were analysed using Rank Products (RP) analysis complete with Bejamini-Hochberg false discovery rate (FDR) adjustment for multiple testing.
FDR <0.05 was considered significant. A minimum individual fold change for significance was not set due to an interest in pathway interactions. Venn diagrams were used to visualize

the results by age and brain section, then Ingenuity Pathway Analysis® (IPA) (Ingenuity Systems, http://www.ingenuity.com) analysed data using both a prespecified candidate gene approach (looking for changes in genes and pathways thought to be relevant from previous work by ourselves and others) and a genome-wide approach (to generate new hypotheses). Significance of pathways was assessed using one-sided Fisher's exact tests.

qRT-PCR data (cycle threshold (CT) values) was analysed in Microsoft® Excel by comparing mean delta cycle threshold (dCT) values ***vs.*** the housekeeper gene using a Student's ***t***-test.

Immunohistochemistry

Tissue preparation: Formalin-fixed tissue was sectioned into 3-mm-thick coronal slices, processed and embedded in paraffin blocks, which were then cut into 7-μm sections. Similar frontal and mid coronal sections were chosen for analysis. "Standard coordinates from a stereotactic atlas were used to identify prominent structures (e.g. the internal capsule). The frontal region encompassed a region approximately +1.8-mm bregma (containing the anterior commissure and anterior lateral ventricle) and the mid coronal captured a region at −1.72-mm bregma (including areas of the basal ganglia and internal capsule)".

Antibody staining: Antibodies assessed various components of the neurovascular unit, claudin-5, collagen IV, smooth muscle actin (SMA), collagen I, glial fibrillary acidic protein (GFAP), matrix metalloproteinase 9 (MMP9), ionized calcium-binding adaptor molecule 1 (Iba-1) and Myelin Basic Protein (MBP). We previously assessed this panel of antibodies in 5 and 16 week old WKY and SHRSP [8]. All immunohistochemical studies used the ABC immunoperoxidase method (Vector Laboratories, Peterborough, UK). Antigen heat retrieval using a pressure cooker (with slides immersed in citric acid buffer) was performed before slides were blocked in hydrogen peroxide followed by either rabbit or swine serum. 3.3′-

diaminobenzidine tetrahydrochloride with a haematoxylin counterstain revealed immunoreactivity. Tris-buffered saline replaced the primary antibody in negative controls.

Data analysis: Percentage staining within a defined area of interest was generated using ImagePro™ software (version 6.2; Media Cybernetics, Bethesda, MD, USA), blinded to species and salt, in cortical, deep grey and white matter. A standard sampling protocol was developed by identifying cross sections using landmarks such as the rhinal fissure and piriform cortex (for full protocol see [8]). Each cross section was divided into cortical grey matter, white matter and deep grey matter using the freehand tool on ImagePro™. Intra-observer reliability was assessed using a randomly selected slide counted on five separate occasions and revealed less than 10% of the variance within the counts was due to the effect of day. Colour-matched pixel counts applied to the entire hemisphere and subsequently converted into percentage areas of staining as a measure of immunoreactivity using ImagePro™, once each region of interest (e.g. cortical grey) had been defined by hand.

We performed statistical analysis in Minitab using a general linear model (two-way ANOVA) followed by Tukeys test for pairwise comparisons. The effect of both salt diet and strain were analysed. Frontal cortex and mid-coronal sections were analysed separately due to significant differences in the distribution of grey and white matter, and the influence of brain territory on expression was also included. P values of $p<0.05$ were considered statistically significant. All data are shown as mean ± SEM.

Table S1. Results of ANOVA analysis on the effect of salt on the percentage staining of 4 antibodies assessing vascular structure.

	SECTION	FRONTAL SECTION					
	SALT	No Salt			Salt		
	REGION	Cortex	White Matter	Deep Grey	Cortex	White Matter	Deep Grey
Claudin5	WKY	0.280±0.123	0.268±0.127	0.231±0.162	0.160±0.063	0.112±0.057	0.085±0.049
	SHRSP	0.132±0.027	0.073±0.045	0.048±0.033	0.104±0.029	0.126±0.048	0.117±0.087
	P for salt	WKY:WKY+NaCl No significant differences		WKY+NaCl:SHRSP+NaCl No significant differences		SHRSP:SHRSP+NaCl No significant differences	
Collagen I	WKY	0.172±0.047	0.134±0.079	0.120±0.072	0.094±0.050	0.080±0.039	0.077±0.051
	SHRSP	0.138±0.100	0.066±0.032	0.030±0.014	0.097±0.064	0.099±0.067	0.030±0.014
	P for salt	WKY:WKY+NaCl No significant differences		WKY+NaCl:SHRSP+NaCl No significant differences		SHRSP:SHRSP+NaCl No significant differences	
Collagen IV	WKY	0.183±0.120	0.234±0.162	0.062±0.017	0.203±0.142	0.106±0.062	0.089±0.025
	SHRSP	0.361±0.074	0.120±0.032	0.100±0.044	0.113±0.053	0.041±0.028	0.098±0.049
	P for salt	WKY:WKY+NaCl No significant differences		WKY+NaCl:SHRSP+NaCl No significant differences		SHRSP:SHRSP+NaCl Cortex p=0.02 White = NS Deep Grey = NS	
SMA	WKY	0.189±0.075	0.100±0.049	0.139±0.043	0.298±0.161	0.087±0.025	0.146±0.056
	SHRSP	0.356±0.121	0.260±0.136	0.199±0.087	0.231±0.027	0.171±0.094	0.139±0.067
	P for salt	WKY:WKY+NaCl No significant differences		WKY+NaCl:SHRSP+NaCl No significant differences		SHRSP:SHRSP+NaCl No significant differences	

Data taken from frontal section tissue in 21 week old SHRSP versus WKY rats fed either a normal diet or salt-loaded with 1% NaCl from the age of 18 weeks. Numbers indicate percentage staining ± the standard error of the mean from n=5 animals. NS = No significant difference.

Table S2. Results of ANOVA analysis on the effect of salt on the percentage staining of 4 antibodies assessing vascular structure.

	SECTION	MID CORONAL SECTION					
	**SALT**	**No Salt**			**Salt**		
	**REGION**	**Cortex**	**White Matter**	**Deep Grey**	**Cortex**	**White Matter**	**Deep Grey**
**Claudin5**	WKY	0.200±0.025	0.261±0.140	0.414±0.125	0.233±0.122	0.144±0.084	0.439±0.222
	SHRSP	0.080±0.070	0.129±0.058	0.113±0.084	0.177±0.103	0.233±0.054	0.223±0.085
	P for salt	WKY:WKY+NaCl No significant differences		WKY+NaCl:SHRSP+NaCl No significant differences		SHRSP:SHRSP+NaCl No significant differences	
**Collagen I**	WKY	0.088±0.064	0.060±0.012	0.732±0.381	0.242±0.150	0.131±0.080	0.475±0.202
	SHRSP	0.308±0.135	0.147±0.081	0.356±0.237	0.154±0.050	0.171±0.138	0.349±0.135
	P for salt	WKY:WKY+NaCl No significant differences		WKY+NaCl:SHRSP+NaCl No significant differences		SHRSP:SHRSP+NaCl No significant differences	
**Collagen IV**	WKY	0.239±0.089	0.265±0.121	0.151±0.018	0.311±0.100	0.116±0.068	0.230±0.153
	SHRSP	0.183±0.112	0.054±0.033	0.146±0.044	0.413±0.198	0.229±0.143	0.239±0.085
	P for salt	WKY:WKY+NaCl No significant differences		WKY+NaCl:SHRSP+NaCl No significant differences		SHRSP:SHRSP+NaCl No significant differences	
**SMA**	WKY	0.292±0.044	0.385±0.183	0.291±0.087	0.262±0.076	0.219±0.092	0.649±0.187
	SHRSP	0.530±0.237	0.517±0.231	0.704±0.352	0.426±0.200	0.403±0.118	0.398±0.252
	P for salt	WKY:WKY+NaCl Cortex = NS White = NS Deep Grey p<0.05		WKY+NaCl:SHRSP+NaCl No significant differences		SHRSP:SHRSP+NaCl No significant differences	

Data taken from mid coronal tissue in 21 week old SHRSP versus WKY rats fed either a normal diet or salt-loaded with 1% NaCl from the age of 18 weeks. Numbers indicate percentage staining ± the standard error of the mean from n=5 animals. NS = No significant difference.

Table S3. Results of ANOVA analysis on the effect of salt on the percentage staining of 4 antibodies assessing the presence / absence of vascular disease.

	**SECTION**	**FRONTAL SECTION**					
	**SALT**	**No Salt**			**Salt**		
	**REGION**	**Cortex**	**White Matter**	**Deep Grey**	**Cortex**	**White Matter**	**Deep Grey**
**MMP9**	WKY	0.349±0.211	0.154±0.101	0.096±0.065	0.132±0.098	0.112±0.073	0.087±0.057
	SHRSP	0.148±0.077	0.143±0.081	0.030±0.023	0.264±0.106	0.248±0.174	0.158±0.132
	P for salt	WKY:WKY+NaCl No significant differences		WKY+NaCl:SHRSP+NaCl No significant differences		SHRSP:SHRSP+NaCl No significant differences	
**GFAP**	WKY	0.914±0.199	1.387±0.254	1.490±0.567	0.493±0.136	1.991±0.230	0.835±0.161
	SHRSP	0.715±0.169	2.419±0.758	1.034±0.304	1.046±0.299	1.840±0.314	1.190±0.139
	P for salt	WKY:WKY+NaCl Cortex p<0.05 White = NS Deep Grey = NS		WKY+NaCl:SHRSP+NaCl Cortex p<0.01 White = NS Deep Grey = NS		SHRSP:SHRSP+NaCl No significant differences	
**IBA-1**	WKY	0.063+0.013	0.109+0.042	0.048+0.013	0.031+0.011	0.054+0.016	0.027+0.010
	SHRSP	0.071+0.035	0.084+0.048	0.052+0.028	0.014+0.002	0.030+0.013	0.051+0.026
	P for salt	WKY:WKY+NaCl No significant differences		WKY+NaCl:SHRSP+NaCl No significant differences		SHRSP:SHRSP+NaCl Cortex p<0.01 White = NS Deep Grey = NS	
**MBP**	WKY	0.477±0.191	1.402±0.611	1.690±0.697	1.990±0.794	0.346±0.117	1.165±0.092
	SHRSP	0.462±0.204	0.632±0.224	0.472±0.286	0.427±0.237	0.561±0.294	1.189±0.290
	P for salt	WKY:WKY+NaCl Cortex p=0.01 White p<0.05 Deep Grey = NS		WKY+NaCl:SHRSP+NaCl Cortex p<0.01 White = NS Deep Grey = NS		SHRSP:SHRSP+NaCl Cortex = NS White = NS Deep Grey = p<0.01	

Data taken from frontal tissue in 21 week old SHRSP versus WKY rats fed either a normal diet or salt-loaded with 1% NaCl from the age of 18 weeks. Numbers indicate percentage staining ± the standard error of the mean from n=5 animals. NS = No significant difference.

Table S4. Results of ANOVA analysis on the effect of salt on the percentage staining of 4 antibodies assessing the presence / absence of vascular disease.

	**SECTION**	**MID CORONAL SECTION**					
	**SALT**	**No Salt**			**Salt**		
	**REGION**	**Cortex**	**White Matter**	**Deep Grey**	**Cortex**	**White Matter**	**Deep Grey**
**MMP9**	WKY	0.141±0.058	0.217±0.013	0.521±0.171	0.162±0.115	0.367±0.225	0.717±0.346
	SHRSP	0.452±0.232	0.207±0.102	0.380±0.196	0.350±0.225	0.119±0.056	0.375±0.182
	P for salt	WKY:WKY+NaCl<br>No significant differences		WKY+NaCl:SHRSP+NaCl<br>No significant differences		SHRSP:SHRSP+NaCl<br>No significant differences	
**GFAP**	WKY	0.683±0.302	2.388±0.529	1.898±0.496	0.615±0.174	2.439±0.382	2.551±0.382
	SHRSP	0.702±0.172	2.093±0.500	2.246±0.515	0.504±0.162	3.965±0.766	2.552±0.702
	P for salt	WKY:WKY+NaCl<br>No significant differences		WKY+NaCl:SHRSP+NaCl<br>Cortex = NS White p<0.05 Deep Grey = NS		SHRSP:SHRSP+NaCl<br>Cortex = NS White p<0.01 Deep Grey = NS	
**IBA-1**	WKY	0.032+0.020	0.022+0.011	0.013+0.006	0.055+0.020	0.034+0.021	0.019+0.009
	SHRSP	0.045+0.015	0.034+0.019	0.036+0.020	0.112+0.033	0.109+0.099	0.063+0.026
	P for salt	WKY:WKY+NaCl<br>No significant differences		WKY+NaCl:SHRSP+NaCl<br>Cortex p<0.05 White = NS Deep Grey p=0.01		SHRSP:SHRSP+NaCl<br>Cortex p=0.01 White = NS Deep Grey = NS	
**MBP**	WKY	4.622±2.152	1.659±0.634	0.582±0.228	3.834±1.410	0.521±0.150	0.292±0.107
	SHRSP	1.570±0.786	1.098±0.761	0.383±0.233	1.944±0.886	0.063±0.046	0.130±0.105
	P for salt	WKY:WKY+NaCl<br>Cortex = NS White = p<0.01 Deep Grey = NS		WKY+NaCl:SHRSP+NaCl<br>Cortex = NS White = p<0.05 Deep Grey = NS		SHRSP:SHRSP+NaCl<br>Cortex = NS White = p<0.05 Deep Grey = NS	

Data taken from mid coronal tissue in 21 week old SHRSP versus WKY rats fed either a normal diet or salt-loaded with 1% NaCl from the age of 18 weeks. Numbers indicate percentage staining ± the standard error of the mean from n=5 animals. NS = No significant difference.

WKY S v NS Frontal					
**Symbol**	**Entrez Gene Name**	**Expr p-value**	**Expr Fold Change**	**Location**	**Family**
ACTB	actin beta	0.002937063	-2.254365334	Cytoplasm	other
AHSG	alpha 2-HS glycoprotein	0.028353808	2.161816113	Extracellular Space	other
ALB	albumin	0.01	2.038292073	Extracellular Space	transporter
AMBP	alpha-1-microglobulin/bikunin precursor	0.011185771	2.61159513	Extracellular Space	transporter
APP	APP	0.041423671	-1.825170257	Plasma Membrane	other
CPE	CPE	0.001363636	-2.381731904	Plasma Membrane	peptidase
MAP1B	microtubule associated protein 1B	0.009760766	-2.191795586	Cytoplasm	other
MBP	myelin basic protein	0.000649351	-2.636272104	Extracellular Space	other
NSF	N-ethylmaleimide sensitive factor, vesicle fusing ATPase	0.039690522	-1.945245839	Cytoplasm	transporter
NTS	neurotensin	0.041423671	1.823815196	Extracellular Space	other
TTR	transthyretin	0.036727273	-1.009580577	Extracellular Space	transporter

SHRSP S vs NS Frontal					
**Symbol**	**Entrez Gene Name**	**Expr p-value**	**Expr Fold Change**	**Location**	**Family**
ACTB	actin beta	0.000363636	3.664505185	Cytoplasm	other
AHSG	alpha 2-HS glycoprotein	0.886386098	1.564397312	Extracellular Space	other
ALB	albumin	0.252661871	2.18686048	Extracellular Space	transporter
AMBP	alpha-1-microglobulin/bikunin precursor	0.420691928	1.687026301	Extracellular Space	transporter
APP	APP	0.229076305	1.867222156	Plasma Membrane	other
CPE	CPE	0.000833333	3.790831982	Plasma Membrane	peptidase
MAP1B	microtubule associated protein 1B	0.00969697	3.039544736	Cytoplasm	other
MBP	myelin basic protein	0.027260442	2.159306134	Extracellular Space	other
NSF	N-ethylmaleimide sensitive factor, vesicle fusing ATPase	0.022399404	2.308452769	Cytoplasm	transporter
NTS	neurotensin	0.552521408	1.529705972	Extracellular Space	other
TTR	transthyretin	0	53.37301373	Extracellular Space	transporter

**SHRSP Salt vs No Salt**					
**Symbol**	**Entrez Gene Name**	**Expr p-value**	**Expr Fold Change**	**Location**	**Family**
ACTB	actin beta	0.000363636	3.664505185	Cytoplasm	other
CAPNS1	calpain small subunit 1	0.017414141	2.132061626	Cytoplasm	peptidase
CTNNB1	catenin beta 1	0.004545455	2.700607847	Nucleus	transcription regulator
DNAJB4	DnaJ heat shock protein family (Hsp40) member B4	0.025200535	2.130853613	Nucleus	other
DSTN	DSTN	0.012545455	2.516480202	Cytoplasm	other
HNRNPA2B1	heterogeneous nuclear ribonucleoprotein A2/B1	0.040969125	1.883773987	Nucleus	other
KCNIP4	potassium voltage-gated channel interacting protein 4	0.029879518	2.234544061	Plasma Membrane	ion channel
MBP	myelin basic protein	0.027260442	2.159306134	Extracellular Space	other
PLS3	plastin 3	0.02084048	2.149291695	Cytoplasm	other
ROCK2	Rho associated coiled-coil containing protein kinase 2	0.021338843	-1.914940256	Cytoplasm	kinase
RPL7	ribosomal protein L7	0.015159705	2.413802708	Cytoplasm	transcription regulator
YAF2	YAF2	0.016807611	2.083912782	Nucleus	transcription regulator
YY1	YY1 transcription factor	0.015159705	2.382734897	Nucleus	transcription regulator
